# Psychometric Evaluation of the Yale-Brown Obsessive-Compulsive Scale Modified for Body Dysmorphic Disorder for Adolescents (BDD-YBOCS-A)

**DOI:** 10.1007/s10578-022-01376-x

**Published:** 2022-06-09

**Authors:** Benedetta Monzani, Deanna Fallah, Daniel Rautio, Martina Gumpert, Amita Jassi, Lorena Fernández de la Cruz, David Mataix-Cols, Georgina Krebs

**Affiliations:** 1 National and Specialist OCD and Related Disorders Clinic for Young People, South London and Maudsley NHS Foundation Trust, London, UK; 2 Institute of Psychiatry, Psychology & Neuroscience, King’s College London, London, UK; 3 Centre for Psychiatry Research, Department of Clinical Neuroscience, Karolinska Institutet, Stockholm, Sweden; 4 Stockholm Health Care Services, Region Stockholm, Stockholm, Sweden

**Keywords:** Body dysmorphic disorder, Dysmorphophobia, Symptom severity, Adolescents, Psychometric evaluation, Factor analysis, Validity, Reliability

## Abstract

The *Yale-Brown Obsessive-Compulsive Scale Modified for Body Dysmorphic Disorder for Adolescents* (BDD-YBOCS-A) is a clinician-rated measure of BDD symptom severity in youth. Despite widespread use in both research and clinical practice, its psychometric properties have not been formally evaluated. The current study examined the factor structure, reliability, validity, and sensitivity to change of the BDD-YBOCS-A in 251 youths with BDD attending two specialist clinics. A principal component analysis identified two factors, explaining 56% of the variance. The scale showed good internal consistency (Cronbach’s alpha = 0.87) and adequate convergent and divergent validity. In a subgroup of participants receiving BDD treatment (*n =* 175), BDD-YBOCS-A scores significantly decreased over time, demonstrating sensitivity to change. BDD-YBOCS-A change scores over treatment were highly correlated with severity changes measured by the Clinical Global Impression – Severity scale (*r* = .84). The study provides empirical support for the use of the BDD-YBOCS-A in children and adolescents with BDD.

## Introduction

Body Dysmorphic Disorder **(**BDD) is a psychiatric condition characterised by a preoccupation with perceived flaws in physical appearance that are not visible or appear slight to others. This preoccupation leads to clinically significant distress and/or impairment in functioning, avoidance, and time-consuming repetitive behaviours or mental acts (e.g., mirror checking, excessive grooming, comparing appearance to others) [1]. Although BDD remains an under-diagnosed and under-studied condition, a systematic review estimated its weighted prevalence to be 1.9% in community adult samples [2]; similar rates have been reported among adolescents, with prevalence estimates ranging from 1.7 to 2.2% in the community [2–5] and up to 14.3% in inpatient adolescent units [6, 7]. BDD is a chronic and often disabling disorder, associated with markedly poor psychosocial functioning and quality of life as well as high rates of suicidality [8, 9]. Encouragingly, meta-analytic evidence shows that Cognitive Behaviour Therapy (CBT) and Selective Serotonin Reuptake Inhibitors (SSRIs) are effective treatments for BDD [10, 11]. Evidence in children and adolescents with BDD is more limited, although also in support of the use of CBT and SSRIs [12–15].

The *Yale-Brown Obsessive Compulsive Scale Modified for BDD* (BDD-YBOCS) [16] is a 12-item, semi-structured, clinician-rated measure of BDD. It is the gold-standard measure used in clinical practice as well as in research, including clinical treatment trials, to assess BDD symptom severity. This scale is a modified version of the original 10-items Yale-Brown Obsessive Compulsive Scale (Y-BOCS) [17], a reliable, valid and widely used measure of Obsessive Compulsive Disorder (OCD) symptom severity. The Y-BOCS scale was chosen for adaptation for BDD on the basis of the many similarities between BDD and OCD, including obsessional thinking and ritualistic behaviours. Like in the Y-BOCS, the first five items of the BDD-YBOCS assess obsessions (in relation to perceived flaw/s in appearance) whilst items 6 through 10 assess compulsive behaviours. Unlike the YBOCS, the BDD-YBOCS includes two additional items measuring insight and avoidance that count towards the total score (see [16] for further details on scale development). The BDD-YBOCS was initially developed and validated to assess adults with BDD; however, the scale has also been adapted for children and adolescents (i.e., BDD-YBOCS-A). The BDD-YBOCS-A maintains the same structure and content of its adult counterpart, the only difference between the two versions of the scale being in the wording; specifically, some of the items were modified to make them age and developmentally appropriate for young people. For example, as opposed to “*How much distress do your thoughts about your body defect(s) cause you?”*, the probe for ‘interference from obsessions’ (item 2) was slightly modified to “*How much do these thoughts about how you look bother or upset you?*” in the adolescent version. The list of ways BDD worries interfere with daily living in adolescents also slightly differs from the adult version of the scale, excluding for example an item on intimacy. Both scales are freely available online.

Three previous studies have examined the psychometric properties of the adult version of the BDD-YBOCS; in all these studies, the BDD-YBOCS demonstrated good reliability, construct validity, and sensitivity to change [16, 18, 19]. However, different factors emerged in the two studies examining its structure. Phillips et al. (1997) (*n =* 125) identified a three-factor solution, reflecting ‘*core symptoms’* (items: 1, 2, 3, 7, 11, and 12), ‘*compulsions’* (items: 6, 7, 8, 9, and 10), and *‘resistance and control of thoughts’* (items: 3, 4, and 5). In another study (*n =* 200), a two-factor structure emerged instead, with one factor including all ‘*core DSM-5 symptoms’* except for interference due to thoughts and avoidance (i.e., items: 2 and 12) [18]. Finally, the Brazilian version of this scale also showed adequate psychometric properties in a sample of adults (*n =* 63), although the authors did not examine its factor structure [19]. Of note, all of these studies primarily focused on adults with BDD; only one [18] included a small sub-sample of adolescents (*n =* 21), representing only 10% of the sample. To the best of our knowledge, the BDD-YBOCS-A remains to be specifically validated in children and adolescents. The lack of data on the reliability and validity of the scale in young people is an important issue to address as the onset of BDD is during adolescence and important clinical and developmental differences between children/adolescents and adults have been observed in the literature [9]. Not least, young people with BDD present with poorer insight compared to adults [20], which may in turn impact on recognition and/or disclosure of the extent and nature of symptoms during the BDD-YBOCS-A interview. Given the increasing efforts and attention in adolescent BDD and the likelihood that the BDD-YBOCS-A will continue to be used by clinicians and researchers in this field, further examination of its psychometric properties in adolescence is warranted.

The aim of the current study was to assess the psychometric properties of the BDD-YBOCS-A in a large, well-characterized sample of children and adolescents with BDD. Based on previous studies, we expected a two- or three-factor solution, mapping on to the BDD diagnostic criteria. It was also hypothesised that, similarly to the original adult version, the scale would have high internal consistency and display higher correlations with other measures of BDD symptoms than with measures not related to body image concerns (e.g., measures of depressive symptoms), and that the instrument would be sensitive to change after multimodal, evidence-based treatment.

### Methods

#### Sample

Participants included 251 youth (aged between 10 and 19) meeting DSM-5 [1] criteria for BDD who were referred to either the National and Specialist OCD, BDD, and Related Disorders Clinic for Young People at the Maudsley Hospital in London, England (*n =* 143) or the OCD and Related Disorders Clinic for Children and Adolescents in Stockholm, Sweden (*n =* 108) between 2011 and 2020. There were no inclusion/exclusion criteria other than having a diagnosis of BDD. A subsample of 175 patients (69.7%) received CBT for BDD according to a validated protocol [13, 15] and/or medication for their symptoms and had post-treatment data available for analysis. The remaining patients (*n =* 76, 30.3%) were either referred elsewhere for treatment or did not have post-treatment data available (e.g., currently in treatment). The study was approved by the South London and Maudsley Child and Adolescent Mental Health Service Audit Committee and by the Regional Ethical Review Board in Stockholm.

#### Measures

The ***Yale-Brown Obsessive-Compulsive Scale Modified for Body Dysmorphic Disorder for Adolescents*** *(BDD-YBOCS-A)* [16] is a 12-item semi-structured, clinician-administered interview that assesses BDD symptom severity. The scale is a modified version of the BDD-YBOCS for adults that was first developed in 1997 by Phillips and colleagues [16] using the Y-BOCS [17] for OCD; the scale has shown excellent interrater and test-retest reliability and good convergent and divergent validity in mostly adult samples [16, 18, 19]. As in the adult version, items 1–5 assess the extent of the preoccupations about physical appearance, items 6–10 assess appearance-related compulsive behaviours, item 11 assesses insight into appearance beliefs, and item 12 assesses avoidance due to BDD symptoms. Each item is rated on a 0–4 scale. The total BDD severity score therefore ranges from 0 to 48, with higher scores reflecting higher symptom severity. The adult version of the scale showed sensitivity to change [16, 18], with treatment response defined as a BDD-YBOCS reduction of ≥30%, and a BDD-YBOCS score ≤16 corresponding to full or partial symptom remission [21].

The ***Children’s Global Assessment Scale*** (*CGAS*) [22] is a clinician-rated measure of global functional impairment associated with psychopathology. Scores range from 1 to 100, with higher scores indicating better functioning. The CGAS has good psychometric properties, with high inter-rater reliability as well as divergent and convergent validity [22].

The ***Clinical Global Impression – Severity*** *scale (CGI-S)* [23] is a single-item clinician-rated measure of the patient’s current illness severity. Scores range from 1 (‘normal, not at all ill’) to 7 (‘amongst the most extremely ill patients’). The CGI-S is a widely used measure in mental health treatment trials [23] that has shown good convergent validity and sensitivity to change [24].

The ***Appearance Anxiety Inventory*** *(AAI)* [25] is a self-reported measure of BDD-related cognitions and behaviours. It consists of 10 items, each scored on a 0–4 Likert scale. Scores range from 0 to 40, with a cut off score ≥ 20 suggesting high probability of clinical problems [26]. The measure includes two subscales: avoidance and threat monitoring. It has been found to have good test-retest reliability and convergent validity in the measurement of appearance anxiety, and correlates moderately well with other measures of appearance concerns, including the adult version of BDD-YBOCS [25, 27].

Depressive symptoms were assessed with different measures across sites. The London clinic site used the depression subscale of the Beck Youth Inventory, namely the ***Beck Depression Inventory for Youth*** (*BDI-Y)* [28] between 2012 and 2015 and the ***Mood and Feeling Questionnaire Child-Version*** *(MFQ-C)* [29] thereafter. The *BDI-Y* is a 20-item self-report measure of depressive symptoms that has demonstrated good internal consistency and test-criterion validity [28]. The MFQ-C on the other hand comprises 33 items, designed to measure depressive symptoms in youth between 6 and 19 years old, with good evidence in favour of its strong psychometric properties [29–31]. The Stockholm site on the other hand used the 10-item ***Children’s Depression Inventory-Short Version*** (*CDI-S)* [32] from 2015 to 2018 and the 13-item ***Short Mood and Feeling Questionnaire Child*** **-** ***Version*** (*SMFQ-C)* [33] from 2018 and onwards. The CDI-S has demonstrated good to excellent psychometric properties [32–34]. The SMFQ-C has shown to be a reliable and valid measure of depression in children in both clinical and non-clinical samples [29–31]. A *z*-score transformation was computed to combine and compare the scores from the various self-report measures of depressive symptoms [35].

#### Procedure

Patients from both clinic sites underwent similar diagnostic assessment procedures. This consisted of a three-hour assessment conducted by a multi-disciplinary team where a series of interviews were completed, including a full psychiatric and developmental history. In Stockholm, this involved the use of the Mini International Neuropsychiatric Interview for Children (MINI-KID) [36], supplemented with additional modules for obsessive–compulsive and related disorders. In London, this was done with the Development and Well-Being Assessment (DAWBA) [37]. The BDD-YBOCS-A interviews were conducted by clinical psychologists with expertise in BDD, assistants or trainee clinical psychologists under close supervision to assure specialist quality assessments. The training procedure for administrating the instrument involved an observation component, followed by joint and then supervised administration of the scale. In addition, instructions with verbatim scripts were available for BDD-YBOCS-A interviews. All BDD-YBOCS-A interviews were discussed with the multidisciplinary team and diagnosis of BDD was confirmed based on the information gained from the BDD-YBOCS-A interview, parental account of current difficulties, and developmental history. Self-reported measures were collected prior to the initial assessment and at post-treatment (see Rautio et al. [15] for further details). A total of 175 patients (69.7%) received CBT and/or medication for BDD; the CBT consisted of weekly sessions incorporating psychoeducation, exposure and response prevention, and relapse prevention, as previously described [13, 15]. Regarding medication for BDD, some patients were already on medication prior to assessment; in other cases, medications were modified following the assessment or during treatment, according to the team’s clinical judgement.

#### Statistical analyses

Principal component analysis (PCA) with varimax rotation was conducted on all BDD-YBOCS-A items to analyse the factor structure of the scale. Eigenvalues greater than 1 and the scree plot were used to identify the number of factors. Internal consistency was evaluated using Cronbach’s alpha coefficient, with a minimal alpha value of 0.70 regarded as acceptable. Reliability assessment also included item-total correlations (ITC). An ITC correlation greater than 0.30 is considered an acceptable contribution of the item to the measure [38].

Convergent and divergent validity were examined using Pearson’s correlation between total BDD-YBOCS-A scores and total scores on other measures, including the CGAS, the AAI, and *z*-transformed self-reported measure of depression. A stronger correlation of the BDD-YBOCS-A with the AAI scores, compared to the CGAS and depression scores was used to provide evidence of convergent/divergent validity.

Finally, to evaluate the sensitivity to change of the BDD-YBOCS-A, we conducted a paired sample *t*-test to calculate the change in scores from pre- to post-treatment for the sub-group of youth undergoing multimodal treatment for BDD (*n =* 176). A significant decrease in the total BDD-YBOCS-A score would indicate that the instrument is sensitive to change. Within-group effect size (Cohen’s *d*) and correlation between changes in BDD-YBOCS-A scores and CGI-S scores pre- to post-treatment would also provide further evidence for the measure’ sensitivity to change.

## Results

### Descriptive statistics

Demographic and clinical characteristics of the sample are presented in Table 1. The majority of the sample were girls (*n =* 201, 80.1%); the age range was 10 to 19 years with a mean age of 15.62 (*SD =* 1.44). The mean BDD-YBOCS-A total score was 32.55 (*SD* = 5.98; range 12–45), whilst the CGI-Severity mean score was 4.84 (*SD* = 0.77; range 2–7), corresponding to moderate levels of symptom severity at baseline.

**Table 1:** Study sample characteristics (*n* = 251)

Variable (*n* available)	Mean	SD	Range
Age at assessment in years (*n* = 246)	15.62	1.44	10–19
BDD-YBOCS-A total score (*n* = 244)	32.55	5.98	12–45
CGAS total score (*n* = 241)	43.29	8.57	23–76
CGI-S total score (*n* = 201)	4.84	0.77	2–7
AAI total score (*n* = 203)	27.33	8.87	2–40
BDI-Y/MFQ-C/CDI-S/SMFQ-C ^a^ total score (*n* = 194)	0.01	0.988	-2.48–1.94

*Abbreviations: BDD-YBOCS-A Yale-Brown Obsessive-Compulsive Scale Modified for BDD for Adolescents*, *CGAS* Children ´s Global Assessment Scale, *CGI-S* Clinical Global Impression – Severity, *AAI* Appearance Anxiety Inventory, BDI-Y Beck Depression Inventory Youth, *MFQ-C* Mood and Feeling Questionnaire-Child Version, *CDI-S* Children’s Depression Inventory – Short Version, *SMFQ-C* Short Mood and Feeling Questionnaire-Child Version, *SD* standard deviation

^a^ Scores on BDI, MFQ-C, SMFQ-C, and the CDI-S were transformed into z-scores for the analysis

### Factor structure

**Fig. 1:**
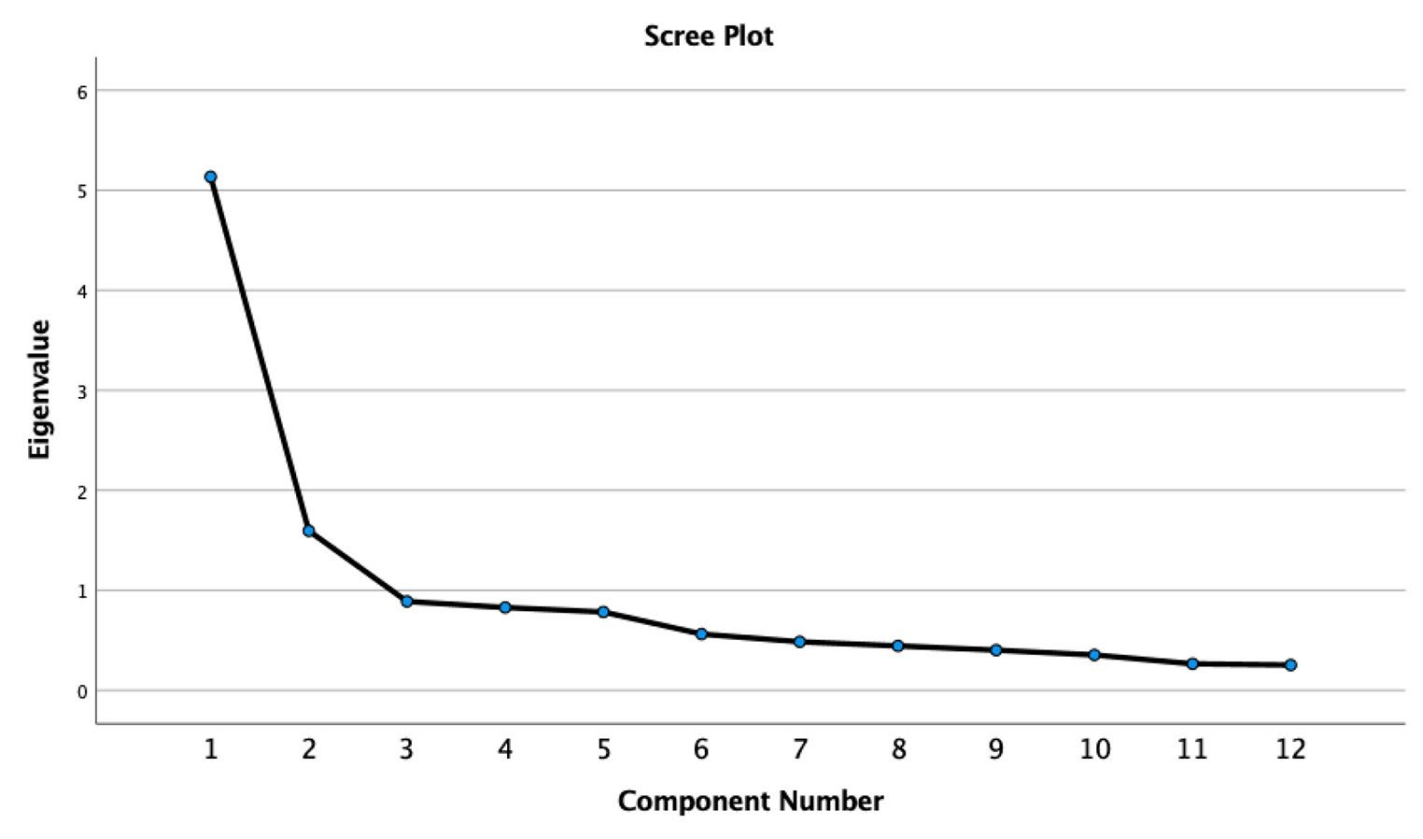
Scree plot for the BDD-YBOCS-A Principal Component Analysis

Bartlett’s Test of Sphericity was significant (χ2 (66) = 1187.20, p < .001), indicating an acceptable number of significant correlations among variables. The Kaiser Meyer Olkin Measure of Sampling Adequacy (KMO) for the overall sample was good (0.85). A two-factor solution was identified based on eigenvalues higher than 1, accounting for 56.1% of the variance; the scree plot was in support of this factor structure (see Fig. 1). The first factor explained 42.8% of the variance. This factor included the time, interference, and distress items for thoughts and behaviours plus the avoidance item, with factor loadings ranging from 0.54 to 0.79 (Table 2). The second factor explained 13.3% of the variance and it included the resistance and control items for thoughts and behaviours in addition to the insight item, with factor loadings ranging from 0.49 to 0.82 (Table 2).

**Table 2:** Mean scores, reliability data, and factor loadings for individual items scores on the BDD-YBOCS-A at baseline (*n =* 251)

Scale Item	Mean	SD	ITC^a^	Cronbach’s Alpha^b^	Factor Loading	
					**Factor 1**	**Factor 2**
*1. Time preoccupied with thoughts*	3.05	0.77	0.56	0.85	0.75	
*2. Interference due to thoughts*	2.52	0.65	0.55	0.86	0.79	
*3. Distress due to thoughts*	2.68	0.683	0.57	0.85	0.75	
*4. Resistance against thoughts*	2.51	0.93	0.46	0.86		0.74
*5. Control over thoughts*	2.91	0.73	0.61	0.85		0.65
*6. Time spend in behaviours*	2.85	0.70	0.61	0.85	0.72	
*7. Interference due to behaviours*	2.37	0.74	0.65	0.85	0.78	
*8. Distress due to behaviours*	2.97	0.66	0.64	0.85	0.55	
*9. Resistance against behaviours*	2.79	0.83	0.45	0.86		0.83
*10. Control over behaviours*	2.88	0.77	0.63	0.85		0.73
*11. Insight*	2.59	0.81	0.40	0.86		0.49
*12. Avoidance*	2.44	0.94	0.58	0.85	0.58	

^a^ *Item-Total Correlation = Correlation coefficient for correlation between item score and total scale score minus the item score*

^*b*^ *Internal Consistency if item deleted*

### Reliability

The Cronbach’s alpha coefficients at baseline (*n =* 234) and post-treatment (*n =* 175) were 0.87 and 0.96, respectively, demonstrating good to excellent internal consistency. Correlation coefficients between each item on the BDD-YBOCS-A and the total score minus that item for the full sample at baseline were positive and greater than 0.30, ranging from 0.40 to 0.65 (Table 2), supporting the adequate contribution of all items to the measure.

### Convergent and divergent validity

With regards to convergent validity, the BDD-YBOCS-A exhibited a significant and positive correlation with the AAI (*r* = .37, *p* < .01). The scale was also significantly and negatively correlated with clinician ratings of impairment using the CGAS (*r* = − .57, p < .01), indicating that higher BDD symptom severity was associated with poorer functioning. At post-treatment, the correlation between BDD-YBOCS-A and the AAI (*r* = .68, *p* < .01) and CGAS (*r* = .67, *p* < .01) were in the large range. When examining divergent validity, a more modest correlation was detected between the BDD-YBOCS-A and the *z*-transformed score of depressive symptoms (*r* = .26, p < .01).

### Sensitivity to change

There was a significant reduction on the BDD-YBOCS-A from baseline to post-treatment from a mean score of 32.20 (*SD* = 5.83) to a mean score of 17.17 (*SD* = 10.33) (*t =* 21.20, *df =* 173, *p* < .01) for youth undergoing multimodal treatment for BDD. The within-group effect size (Cohen’s *d*) for the BDD-YBOCS-A from baseline to post-treatment was 1.60 (95% CI 1.38–1.83). Furthermore, the change in BDD-YBOCS-A scores from baseline to post-treatment was highly correlated with CGI-S change scores (*r =* .84, p < .01).

## DISCUSSION

The current study represents the first formal psychometric investigation of the adolescent version of the BDD-YBOCS, a tool that is widely considered to be the gold-standard measure of BDD symptoms. Four key findings emerged.

First, we established a two-factor structure for the BDD-YBOCS-A, with all items loading substantially onto one of the following two factors (Table 2). The first factor included items relating to the core DSM-5 criteria for BDD (i.e., time, distress, and impairment caused by obsessional thoughts and repetitive behaviours as well as avoidance). This is broadly in keeping with a previous study that identified a ‘core symptoms’ factor of the BDD-YBOCS in adults [16]. However, it is of note that in this previous study only obsession items loaded on the core symptoms factor, which is in contrast to the current findings where both obsession and compulsion items contributed to our first factor [16]. The second factor included insight, resistance, and control. This grouping of items seems logical, because patients who have greater insight into the excessive and irrational nature of their appearance concerns and related behaviours are more likely to attempt to resist them, and in turn are more likely to experience some degree of control. Although our findings do not directly replicate the factor structure found in adult samples [16, 18], it should be noted that no two previous studies have yielded identical results. Variation in findings may be attributable to difference in sample characteristics and statistical methods employed, and highlight the need to be cautious in generalising findings.

Second, consistent with previous studies in adult samples [16, 18, 19], our results demonstrated that the BDD-YBOCS-A has good to excellent internal consistency. Furthermore, each item of the BDD-YBOCS-A was positively correlated with the total score minus that item, thereby providing further evidence that it is a cohesive scale. The lowest correlation of any individual item with the total score minus that item was observed for insight (*r* = .40) and resistance to thoughts (*r* = .45), which replicates the results of a previous study in adults with BDD [18]. This finding is consistent with the notion that insight and efforts to ignore/resist appearance worries are a feature of BDD but are not one of the core symptoms, according to diagnostic criteria [1].

Third, we found evidence to support the convergent and divergent validity of the BDD-YBOCS-A. As expected, the instrument showed a significant and positive correlation with the AAI, a self-report questionnaire of BDD symptoms. The fact that we observed only a modest correlation between the BDD-YBOCS and the AAI may be due to the fact that the two measures are completed by different informants (i.e., clinician-report versus self-report). Previous studies in adults have reported substantially higher correlations between the BDD-YBOCS and another clinician-administered measure of BDD [18, 19], suggesting that higher agreement may be observed within informant. In keeping with this, in the current study the BDD-YBOCS-A showed a stronger correlation with the CGAS (*r* = .60), a clinician-administered measure of global functioning. Conceptually, we would expect these two measures to be associated, but the fact that this was the highest correlation may be attributable to common method variance (i.e., both measures were completed by the same person). Interestingly, the correlation between BDD-YBOCS-A and the AAI was higher at post-treatment (*r* = .68) and comparable to the CGAS (*r* = .67), providing more robust evidence of convergent validity, as well as raising the possibility that the lower correlation at baseline could also be attributable to poorer insight/awareness when reporting BDD symptoms at baseline, compared to post-treatment. As expected, we found that the BDD-YBOCS-A was most weakly correlated with self-reported depression, thereby providing evidence of divergent validity. The fact that this correlation was still significant is in keeping with previous research showing that BDD and depressive symptoms commonly co-occur [9].

Fourth, in keeping with previously reported findings examining the psychometric properties of the scale in adult samples [16, 18], we found that BDD-YBOCS-A scores decreased significantly from baseline to post-treatment, demonstrating sensitivity to change. The high and significant correlation between the BDD-YBOCS-A and CGI-S changes in scores from baseline to post-treatment (*r* = .84) is in further support of the scale’s ability to sensitively measure changes in symptom severity after treatment. Taken together, the BDD-YBOCS-A appears to be an appropriate measure for evaluating treatment outcomes among young people with BDD, supporting its continued use in both research trials [13, 14] and clinical settings [15].

The current study has several notable strengths, including its large sample size of well-characterized cases (*N =* 251), good distribution of participant ages capturing the breadth of youth (10–19 years), and diverse sample recruited from two European countries. However, there are several limitations that should be considered. The majority (80%) of participants in our study were girls. This is consistent with several previous studies showing a female preponderance among youth with BDD in clinical settings [13, 20, 39]. Nevertheless, given that there are certain differences in the clinical presentation of BDD in boys versus girls [9], future studies should seek to include a greater proportion of boys and to stratify analyses by gender. We did not have information on the sample’s ethnic composition, which means that further work will be needed to evaluate the suitability of the scale across different ethnic groups. In addition, our study sample comprised patients with relatively severe BDD symptoms, reflecting the services from which they were drawn. Indeed, it is important to note that patients in this study came from specialist clinics which typically see highly comorbid and treatment-resistant cases (see Rautio et al. [9, 15] for further details) and therefore may limit the generalisability of the study findings. Also of note, although assessments were carried out by clinicians with extensive knowledge of the disorder and trained in the administration of the scale, the measure was used by different personnel across two separate sites. Hence, inter-rater differences cannot be fully ruled out and future research would benefit from assessing inter-rater reliability for the BDD-YBOCS-A. However, taken together, and encouragingly, our findings are broadly consistent with those found in adult samples across different settings, including psychiatric and cosmetic surgery clinics [16, 18, 19]. Lastly, whilst the BDD-YBOCS-A (and its adult counterpart) remain the gold standard measure for assessing BDD symptom severity, these scales are based on the original Y-BOCS [17] which was revised (i.e., YBOCS-II [40]) to overcome various psychometric limitations. Whether similar adaptations will be needed for the YBOCS-BDD and YBOCS-BDD-A should be explored.

### Summary

This study was the first formal evaluation of the BDD-YBOCS-A. A range of psychometric properties were examined in a large sample of young people with BDD attending two specialist services. We found that the scale has strong internal consistency, a two-factor structure, good convergent and divergent validity, and is highly sensitive to change after treatment. Taken together, the study shows that the BDD-YBOCS-A retains the strong psychometric properties of the adult version, supporting its use in both research trials and routine clinical practice with children and adolescents.
